# Effects of maize resistance and leaf chemical substances on the structure of phyllosphere fungal communities

**DOI:** 10.3389/fpls.2023.1241055

**Published:** 2023-08-14

**Authors:** Kun Luo, Gonghua Zhao, Mengfei Chen, Xueliang Tian

**Affiliations:** ^1^ Hunan Agricultural University, Changsha, Hunan, China; ^2^ Henan Engineering Research Center of Biological Pesticide & Fertilizer Development and Synergistic Application, Henan Institute of Science and Technology, Xinxiang, Henan, China

**Keywords:** phyllosphere, fungal community, maize variety, co-occurrence network, *Exserohilum turcicum*, leaf chemical constituents

## Abstract

It is well known that plant genotype can regulate phyllosphere fungi at the species level. However, little is known about how plant varieties shape the fungal communities in the phyllosphere. In this study, four types of maize varieties with various levels of resistances to *Exserohilum turcicum* were subjected to high−throughput sequencing to reveal the properties that influences the composition of phyllosphere fungal communities. The dominant fungi genera for all four maize varieties were *Alternaria* at different relative abundances, followed by *Nigrospora*. Hierarchical clustering analysis, non-metric multidimensional scaling and similarity analysis confirmed that the fungal communities in the phyllosphere of the four varieties were significantly different and clustered into the respective maize variety they inhabited. The findings from Redundancy Analysis (RDA) indicated that both maize resistance and leaf chemical constituents, including nitrogen, phosphorus, tannins, and flavonoids, were the major drivers in determining the composition of phyllosphere fungal communities. Among these factors, maize resistance was found to be the most influential, followed by phosphorus. The co-occurrence network of the fungal communities in the phyllosphere of highly resistant variety had higher complexity, integrity and stability compared to others maize varieties. In a conclusion, maize variety resistance and leaf chemical constituents play a major role in shaping the phyllosphere fungal community. The work proposes a link between the assembled fungal communities within the phyllosphere with maize variety that is resistant to pathogenic fungi infection.

## Introduction

The phyllosphere is a habitat for a diverse collection of microbes including bacteria, fungi and other microorganisms (Jumpponen and Jones, 2009; Rodriguez et al., 2009; Redford et al., 2010). Bacteria are the most abundant microbes in the phyllosphere and play an important ecological role for plants (Bashir et al., 2022). Next to bacteria, phyllosphere fungi form a diverse population that included symbionts, probiotics, and pathogens (Jones and Dangl, 2006; Chaudhary et al., 2017). They also have a vital influence on plants, and even ecosystem functioning (Laforest-Lapointe et al., 2017; Chen et al., 2020b; Liu et al., 2020). For example, phyllosphere fungi can protect host plants from pathogen damage (Arnold et al., 2003), promote plant resistance to environmental stress (Peñuelas et al., 2012) or enhance tolerances towards herbivores by producing toxic alkaloids (Wilkinson et al., 2000; Hartley and Gange, 2009; Estrada et al., 2013). They also influence the dynamics of other taxonomic groups, such as phyllosphere bacteria (Suda et al., 2009), phytophagous insects and their parasitoids (Omacini et al., 2001). They promote the initial decomposition of leaves following senescence (Voriskova and Baldrian, 2013; Kembel and Mueller, 2014), thus contributing to nutrient cycling as early colonizers of leaf litter (Osono, 2006).

Phyllosphere fungi are exposed to the external environment, thus they are constantly exposed to nutrient limitations, UV radiation, and fluctuations in humidity and temperature (Remus-Emsermann and Schlechter, 2018). These fungi depend on the limited amount of nutrients, which are mainly derived from leaves exudates (Inácio et al., 2002). These nutrients contain carbohydrates, amino acids and organic acids and depend on plant species and leaf features such as wettability, waxiness and age (Tukey, 1970). Phyllosphere fungi are also affected by climate (Peñuelas et al., 2012), geographical location (especially at different elevations) (Cordier et al., 2012) and plant genotype (Whipps et al., 2008). For example, the genotype of *Populus balsamifera* located in different geographical regions affected the structure of fungal communities in the phyllosphere (Bálint et al., 2013). Furthermore, plant species and their characteristics were reported to shaped the phyllosphere fungal community structures in a tropical rainforest (Kembel and Mueller, 2014). In addition, the species and cultivar of cereals also influence the phyllosphere fungal communities (Sapkota et al., 2015). Collectively, these studies demonstrated the effect of plant genotype (species, cultivar and regional subpopulations) on the fungal communities of the phyllosphere. Plant resistance to pathogens is also controlled by plant genotype and may shape the microbial community in rhizospheres or phyllospheres. The common bean plant with its high resistance to *Fusarium oxysporum* can alter microbial assemblage in the rhizosphere and enrich biocontrol bacteria (Mendes et al., 2018). Different maize varieties with different resistances to fungal foliar disease can significantly affect the bacterial communities in a phyllosphere (Balint-Kurti et al., 2010). Nevertheless, how plant resistance to fungal pathogens affects the fungal community in a phyllosphere remains unclear.

Maize (*Zea mays* L.) is the most widely grown crop in the world and serves as food, feed, biofuel, and industrial products. In China, more than 35,445 million hectares of land are dedicated to maize cultivation (NBSC, 2017), thus providing an abundance of maize leaves for microbes colonization. Previous studies demonstrated that the maize genotype had a close relationship with the variety of microbes in the phyllosphere. Furthermore, maize genetics influenced the bacterial taxa and metabolic functions of maize leaf microbiomes (Wallace et al., 2018). The variety of maize that is highly resistant to Southern leaf blight had a highly diverse phyllosphere bacterial diversity community (Balint-Kurti et al., 2010). Maize resistance and leaf chemical constituents were noted to jointly shape the bacterial community in a phyllosphere (Tian et al., 2020). These studies showed that maize genotype can structure the bacterial community, but little is known about how maize genotypes (or varieties) influence the structure of the fungal assemblages in a phyllosphere.

To reveal the effect of maize variety on the fungal community in a phyllosphere, we used high-throughput sequencing to analyze the composition and differences within the fungal community associated with four maize varieties that exhibited different resistance to *Exserohilum turcicum*, the causative agent of northern corn leaf blight (NCLB) Moreover, the effect of leaf chemical components on the fungal community was also evaluated. This work contributes to a deeper understanding of the assemblage and function of the fungal community in maize phyllospheres.

## Materials and methods

### Experimental design and planting of maize varieties

Four maize varieties planted in China were selected and classified into four groups based on resistance to *E. turcicum* (highly resistant variety (HR): Zhengyu 8; resistant variety (R): Liaodan 527; susceptible variety (S): Zheng 58; highly susceptible variety (HS): Shendan 16) (Dong et al., 2014). The field experiment was carried out in Xinxiang, Henan Province, the largest summer-maize producing area in China. Northern leaf blight of corn caused by *E. turcicum* is an annual problem occurring in the local fields. Maize varieties were sown on 13 June 2018 and subjected to standard agricultural management. There were three replicate plots for each maize variety and each replicate plot contained 50 maize plants.

### Phyllosphere fungi collection and disease index investigation

Maize leaf samples were collected on 15 August 2018. Healthy ear leaves from ten maize plants of each variety from each plot were randomly selected. Five leaves were used to collect phyllosphere fungi and the other five leaves were used to assess the content of the chemical constituents present in the leaves. The phyllosphere fungi collection was performed as the method of Yao et al. (Yao et al., 2019), with slight modification. In brief, five leaves were cut, rolled, placed into a collection tube and submerged in buffer (0.2 mM Tris, pH 7.5, 0.02 mM EDTA). After vigorous shaking for 5 min, leaves were removed, and the suspension containing phyllosphere fungi was retained. The suspension was filtered through sterile cellulose acetate filters (0.02 μm) to enrich fungi; the filters were placed into the sterilized 50 mL tubes and washed with the Tris-EDTA. The wash eluates were centrifuged at 12, 000 g for 2 min to precipitate the phyllosphere fungi and used to DNA isolation.

The sampling of maize leaves was carried out before any symptoms of NCLB were observed. In order to confirm the resistance of the four maize varieties, we further examined the occurrence of NCLB on an additional thirty maize plants that were naturally infected, following the leaf sampling done 30 days ago. The disease index was calculated according to the published criteria (Wang et al., 2010). To determine if there were significant differences in disease index among the four maize varieties, an analysis of variance (ANOVA) was conducted.

### Leaf chemical characteristics

As previously mentioned, five leaves were collected randomly to assess the content of the chemical constituents’ present. The leaves were washed with sterilized water to remove any dust on the leaf surface and dried in an oven at 60 °C for 48 h after which, the leaves were ground into powder. The nitrogen, phosphorus and soluble sugars contents in the maize leaves were determined according to the methods of Zou et al. (Zou, 2000). Leaf nitrogen was measured by the semi-micro Kjeldahl method. Phosphorus content was determined by the molybdenum blue method. Soluble sugars were extracted from of the dry leaf powder with 60% v/v ethanol and assayed using the phenol–sulfuric acid method. The flavonoid content was determined using the Plant Flavonoids Test Kit (Beijing Baiolibo Technology Co., Ltd., Beijing, China) following the manufacturer’s instructions (Li et al., 2021). Tannin content was determined with the modified colorimetric method (Elemosho et al., 2021). Any significance in the differences between the leaf chemical contents among the four maize varieties was tested using ANOVA analysis.

### High−throughput sequencing

To remove the dust contamination on leaf surface, fungal DNA extraction was performed using the MB Phyllosphere Genomic DNA Kit (MoBio Laboratories, Carlsbad, CA, USA) according to the manufacturer’s instructions. Phyllosphere fungi genomic DNA concentrations were measured with a Nanodrop 3300 spectrophotometer (Thermo Scientific, Wilmington, USA) according to the manufacturer’s instructions. Only DNA samples with the required quality can be used for PCR amplification.

Internal Transcribed Spacer (ITS) fragments were adopted as marker genes and were amplified by Polymerase Chain Reaction (PCR) on the GeneAmp 9700 (ABI, USA) thermocycler for barcoded pyrosequencing using the primers ITS1F and ITS2R (Adams et al., 2013). The primer sequences were ITS1F: 5’-CTTGGTCATTTAGAGGAAGTAA-3’ and ITS2R: 5’-GCTGCGT TCTTCATCGATGC-3’. PCR conditions were set at 95˚C for 2 min (one cycle), 95˚C for 30 s, 55˚C for 30 s, and 72˚C for 30 s (25 cycles), and 72˚C for 5 min (one cycle). The final volume of the PCR reactions was 20 μL which contained 4 μL of 5 × FastPfu Buffer (Promega, USA), 2 μL of 2.5 mM dNTPs, 0.8 μL of each primer (5 μM), 0.4 μL of FastPfu Polymerase (Promega, USA) and 10 ng of template fungal genomic DNA. The PCR products were purified using the AxyPrep DNA Gel Extraction Kit (Axygen Biosciences, Union City, CA, USA) and quantified using QuantiFluorST (Promega, USA). Purified PCR products were pooled in equimolar amounts and paired-end sequenced (2 × 300 bp) Majorbio Bio-Pharm Technology Co. Ltd. (Shanghai, China) on an Illumina MiSeq platform (Illumina, San Diego, USA) according to the standard protocols.

### Bioinformatics processing and data analysis

Bioinformatics analysis was performed on the Majorbio I–Sanger Cloud Platform (https://www.i-sanger.com/). Firstly, the raw sequencing reads were processed using the Quantitative Insights Into Microbial Ecology (QIIME) package (v1.8) (Caporaso et al., 2010). Low-quality sequences, such as primer and barcode sequence mismatches, sequences length < 50 bp, ambiguous bases, PCR-based or sequencing errors and chimeras, were removed. The remaining high-quality sequences were used to produce operational taxonomic units (OTU) by UNITE (https://unite.ut.ee/) with a threshold of 97% identity (Nilsson et al., 2019). Rarefaction curves with average number of observed OTUs were generated to compare relative levels of OTU diversity in the four fungal communities. Shannon index and Simpson index were calculated to assess the α-diversity of the fungal communities. To identify the fungal community composition within the phyllosphere, relative abundance of fungi was assessed at the class and genus levels. The number of common and maize variety-specific fungi were enumerated and presented as a Venn diagram.

Hierarchical clustering analysis, non-metric multidimensional scaling (NMDS) and analysis of similarities (ANOSIM) were performed to reveal the differences among the four varieties using the R 3.1.1 statistical software (Chao et al., 2005; Rajilić-Stojanović et al., 2011; Yu et al., 2016). Linear discriminant Analysis Effect Size (LEfSe) software (Segata et al., 2011) was used to screen for the markedly different genera among the four maize varieties. The Redundancy Analysis (RDA) component in the R package vegan (https://cran.r-project.org/web/packages/vegan/) was used to determine the effect of maize resistance, nitrogen, phosphorus, soluble sugars, tannin and flavonoids on the fungal community in the phyllosphere across all four maize varieties (Oksanen et al., 2012). The two-factor correlation network and linear regression analysis were used on the Majorbio I-Sanger Cloud Platform to establish the relationship between leaf chemical constituents and beta-diversity metrics. To visualize the network structure for the phyllosphere fungi, a network based on the top 50 OTUs was drawn using the Networkx software on the Majorbio I-Sanger Cloud Platform (Zhao et al., 2016). The network parameters such as degree, clustering, degree centrality and closeness centrality were assessed to analyze the complexity of the network. The degree in a network diagram refers to the number of nodes connected to a particular node, indicating its level of connectivity. Clustering represents the interconnectedness of neighboring nodes to a specific node. Degree Centrality is a straightforward measure of node centrality used in network analysis. Closeness centrality is being used to measure the average distance of a node to any other node in the network.

## Results

### Composition of the fungal community in phyllosphere

**Figure 1:**
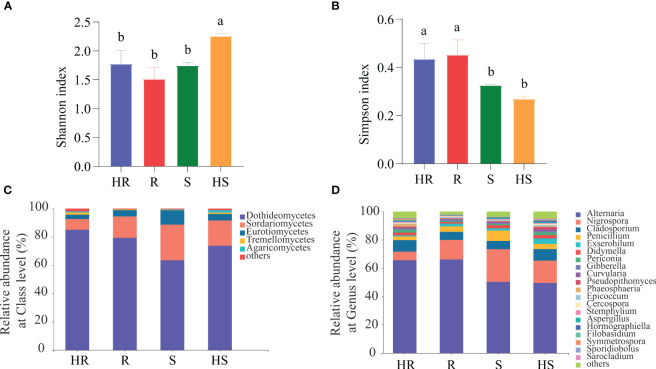
Diversity index and relative abundance of fungi taxa at class and genus level. **(A)** Shannon index. **(B)** Simpson index. **(C)** Relative abundance of fungi at the class level. **(D)** Relative abundance of fungi at the genus level. The ‘others’ in **(D)** represents the combined relative abundance of fungi that are less than 0.1%. Different lowercase letters in **(A, B)** indicated significant differences among four maize varieties (P < 0.01).

High quality ITS fragments sequences were obtained and deposited into the Sequence Read Archive (Accession Number PRJNA871799). Rarefaction curves analysis confirmed that the number of observed OTUs increased asymptotically with an increase in reads (**Supplementary Figure S1A**), indicating that the sequencing and sampling depths were sufficient to cover the diversity. Additionally, both HS and HR had a higher number of observed OTUs in their fungal communities compared to R and S. In terms of alpha-diversity, the Shannon index of the fungal community associated with HS was the highest among all varieties (**Figure 1A**), whereas the Simpson index of the fungal community associated with HR and R was higher than that of S and HS (**Figure 1B**). Based on the taxonomy and abundance of the OTUs, the composition of the fungal communities in the phyllosphere was revealed. At class level, 12 classes of fungi were identified in all four maize varieties (**Supplementary Figures S1B**). The dominant fungi were Dothideomycetes with different relative abundance (HR, 84.9%; R, 79.3%; S, 63.4%; HS, 73.7%) among the four maize varieties, followed by Sordariomycetes (HR, 6.1%; R, 13.3%; S, 24.5%; HS, 17.9%) (**Figure 1C**). At the genus level, 92 genera were identified from all four maize varieties and are proposed as the core fungi (**Supplementary Figure S1C**). The dominant fungus was *Alternaria* with different relative abundance (HR, 65.6%; R, 66.1%; S, 50.3%; HS, 49.6%), followed by *Nigrospora* (HR, 6.1%; R, 13.3%; S, 23.1%; HS, 15.8%) (**Figure 1D**). Some maize pathogen, such as *Exserohilum*, *Gibberella*, *Curvularia*, *Cercospora*, *Bipolaris* and *Ustilago*, were founded. In particular, the relative abundance of the pathogen Northern leaf blight of corn (*Exserohilum*) was significantly higher than that of others maize varieties (**Supplementary Figure S2**). Additionally, nonpathogenic yeasts including *Sporidiobolus* and *Cryptococcus* were also observed. The relative abundance of others fungi in HR was 1.76%, in HR was 0.75%, in S was 1.38%, and in HS was 2.19% (**Figure 1D**).

### Beta diversity analysis

**Figure 2:**
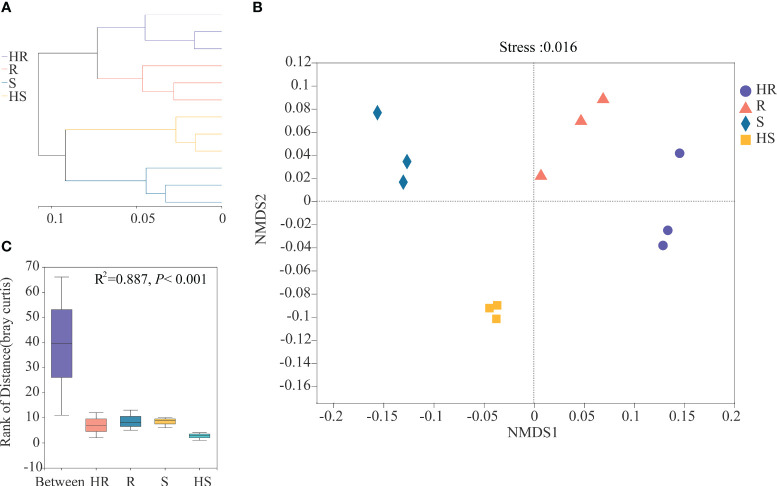
Beta-diversity of the fungal phyllosphere community of the four maize varieties. **(A)** Hierarchical clustering analysis. **(B)** NMDS. **(C)** ANOSIM.

Hierarchical clustering analysis based on Bray-Curtis distance dissimilarities revealed that the fungal communities in the phyllosphere of four maize varieties clustered within the two root branches where HR and R strains were located together and S clustered together with HS (**Figure 2A**). On the sub-branch, the four varieties were separated, suggesting that the fungal communities were different. NMDS, based on the composition of OTUs, revealed that the fungal communities were clustered into four groups consistent with the four maize varieties (**Figure 2B**). The fungal communities in the phyllosphere of HR and R varieties were located at upper right of the Figure. The fungal communities in the phyllosphere of S and HS varieties were clustered towards the left of the figure. ANOSIM also confirmed that the differences in the phyllosphere fungal communities among the four varieties were significant (R^2 = ^0.887, *P* < 0.001) (**Figure 2C**).

**Figure 3:**
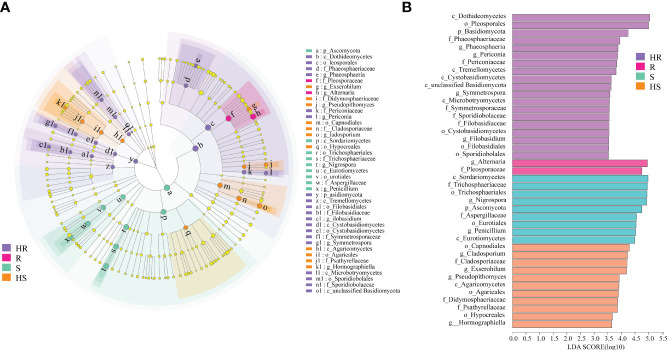
Biomarker taxa identification of the fungal communities in the phyllosphere. **(A)** LefSe analysis. The cladogram shows the taxa with marked differences in the fungal communities in the phyllosphere of the four maize varieties. Yellow nodes represent taxa with no significant difference. Others color nodes indicate markedly different groups with the classification of taxa at the level of phylum, class, order, family, and genus shown from the inside to the outside. **(B)** Species with the significantly higher LDA score compared to the estimated value; the default score is 3.0. The height of the histogram represents the LDA score; i.e., the degree of influence of taxa with a significant difference between distinctive groups.

LEfSe analysis was conducted to reveal which fungi could be the biomarker taxa contributing to differences in the phyllosphere of four maize varieties. The markedly different fungal genera among the four varieties were *Phaeosphaeria*, *Periconia*, *Symmetrospora* and *Filobasidium* in the phyllosphere of the HR variety, *Alternaria* in the phyllosphere of the R variety, *Nigrospora* and *Penicillium* in the phyllosphere of the S variety, and *Cladosporium*, *Exserohilum* and *Hormographiella* in the HS variety phyllosphere (**Figures 3A, B**).

### Effect of maize resistance and leaf chemical constituents on the fungal community

**Figure 4:**
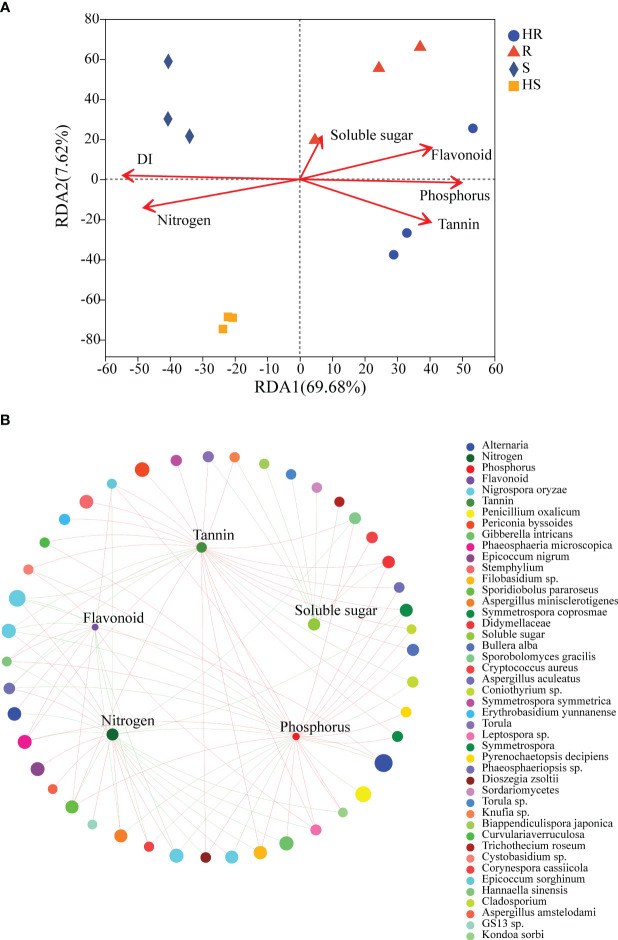
RDA and two-factor correlation network revealed the influence of disease index and leaf chemical constituents on the fungal communities. **(A)** RDA. **(B)** Two-factor correlation network. Red lines represent positive correlations and green lines represent negative correlations. For the two-factor correlation network, a red line indicates a positive correlation, and a green line indicates a negative correlation. DI represents disease index.

The disease index of the four maize varieties exhibited significant differences. HS showed the highest disease index, followed by S and R, whereas HR displayed the lowest disease index (**Supplementary Figure S3A**). The disease index effectively reflects the resistance levels of the four maize varieties. The nitrogen content in S and HS leaves were markedly higher than that of R and HR (**Supplementary Figure S3B**), while the phosphorus, tannin and flavonoid content of S and HS leaves were significantly lower than R and HR (**Supplementary Figures S3C–E**). There was no difference in the content of soluble sugars among the four varieties (**Supplementary Figure S3F**). The RDA results demonstrated that disease index, nitrogen, phosphorus, tannins and flavonoids were the main factors in structuring the fungal communities within a phyllosphere. Phosphorus, tannins and flavonoids were closely related to the fungal communities of HR and R, whereas disease index and nitrogen was correlated to the fungal communities of S and HS (**Figure 4A**). Linear regression demonstrated that disease index had the highest R-value than others leaf chemical substances (**Supplementary Table S1**), proposing that disease index (as well as genotype) was the most important factor for structuring the fungal community associated with the maize varieties. The two-factor correlation network showed that phosphorus, nitrogen, flavonoids and tannins were more positively or negatively correlated with fungi OTUs (**Figure 4B**).

### Fungal community network

**Figure 5:**
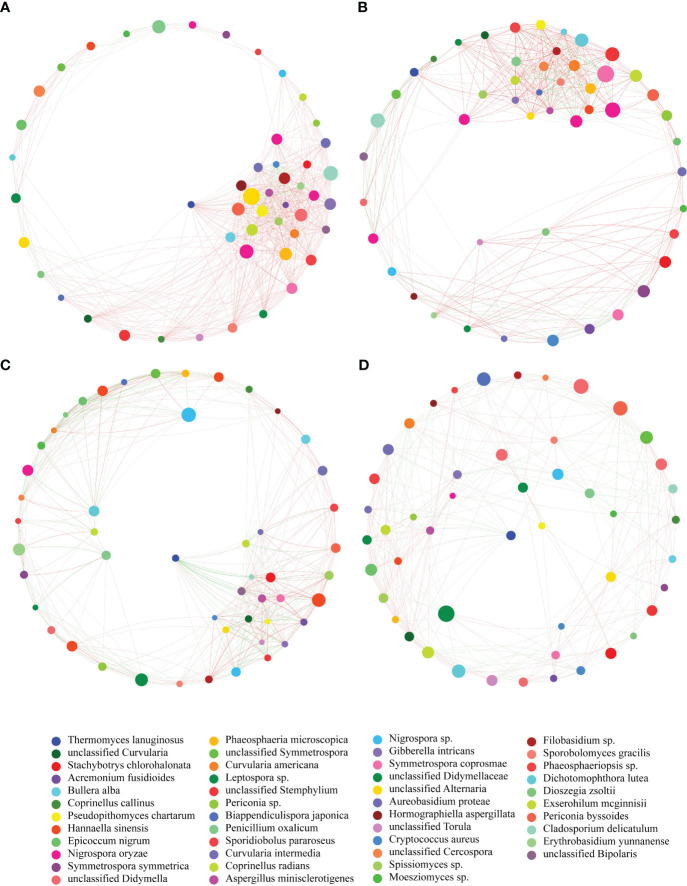
Co-occurrence networks of the fungal communities in the phyllosphere of four maize varieties. Each node represents a fungal OTU and the size of the node is proportional to the number of reads. The color of each node represents the taxonomy of fungi at genus level. The color of each link reflects positive (red) or negative (blue) associations. **(A)** HR. **(B)** R. **(C)** S. **(D)** HS.

The network of fungal communities in the phyllosphere of four maize varieties demonstrated distinct co-occurrence patterns (**Figures 5A–D**). The fungal communities of HR and R had more links between nodes indicating a positive relationship in the network. Moreover, some network parameters, such as degree, clustering, degree centrality and transitivity, were different among the fungal communities of the four maize varieties. The HR fungal community had a higher value in terms of degree and degree centrality (**Supplementary Figures S3A, B**), suggesting that it possessed more connections in the network. Additionally, the HR fungal community also had greater clustering and closeness centrality (**Supplementary Figures S3C, D**), demonstrating that it had superior integrity and stability of the network. Collectively, the fungal communities of HR and R had several positive associations, while S and HS had more negative relationships between fungi identified in the phyllosphere.

## Discussion

In this study, high-throughput sequencing identified a high level of fungal diversity in the phyllosphere of maize leaves. Among the taxa, we identified a variety of filamentous fungi and nonpathogenic yeasts. Most of these fungal taxa were cosmopolitan, as they were commonly found across different plant species. The dominant fungi at the class level were Dothideomycetes and Sordariomycetes, both members of the phylum Ascomycota. This finding is similar to previous reports that Ascomycota was the most common phyllosphere fungi in many crops (Angelini et al., 2012; Janakiev et al., 2019; Ding et al., 2022). At the genus level, the main fungal group was *Alternaria* with different abundance among the four maize varieties, which is similar to the results that *Alternaria* dominate in phyllosphere fungal community of three maize cultivars with different production traits (Kong et al., 2020). The *Alternaria* fungi, as saprophytes or pathogens (Lee et al., 2015), can colonize the phyllosphere of multiple plants (Chen et al., 2020a; Liu et al., 2022), as it can utilize plant debris and organic matter present on the leaves as a source of nutrients (Song et al., 2010). In our study, *Alternaria* in the phyllosphere of maize is assumed to be a saprophyte as no associated disease symptoms were observed on maize leaves. Moreover, *Alternaria* as the dominant fungi on maize phyllosphere probably sustained the stability of the fungal community associated with maize. *Nigrospora* was the second dominant fungal group in the phyllosphere of all four maize varieties. It is well known that *Nigrospora* are widely distributed in various environments and are endophytes or pathogens of plants (Thanabalasingam et al., 2015; Liu et al., 2021). In our study, *Nigrospora* was not a maize-specific pathogen, but rather a saprophyte similar to *Alternaria* in the phyllosphere of maize. In addition, some *Nigrospora* species can produce secondary metabolites that have antifungal and antimicrobial properties, which can help protect the plant from harmful microorganisms (Lu et al., 2022). Further research is needed to determine whether Nigrospora exhibits biocontrol activity in our study.

Of note, *Exserohilum* was found in the four maize varieties, which was not surprising as significant northern leaf blight was observed in our experimental field. Moreover, the *Exserohilum* was the biomarker taxa and had the highest relative abundance in the fungal community of the HS varieties, suggesting that *Exserohilum* survived easily on the leaves of the HS maize variety. Additionally, several common pathogenic fungi associated with maize, such as *Gibberella* (ear rot), *Curvularia* (leaf spot), *Cercospora* (grey leaf spot), *Bipolaris* (southern blight) and *Ustilago* (common smut), were found to have relatively high abundances. This finding indicates that these diseases are commonly observed in local maize production systems. Nonpathogenic yeasts, such as *Sporidiobolus* and *Cryptococcus*, were observed not only on maize leaf surfaces but also on other crops (Sapkota et al., 2015; Chen et al., 2020a), indicating their universal distribution.

In terms of Beta diversity, the fungal communities were not only different among the four maize varieties, but also grouped according to the four resistance levels, reflecting that maize resistance to *Exserohilum* may shape the fungal communities. Our four maize varieties were planted in a one field to reduce any environmental influence on the fungi in the phyllosphere. As previously mentioned, some plants such as poplar (Bálint et al., 2013), trees in rainforest (Kembel and Mueller, 2014) and cereal (Sapkota et al., 2015) can structure the fungal community in the phyllosphere at the plant species level. At the cultivar level, grape plants are important in shaping the phyllosphere fungal community (Bokulich et al, 2014). Moreover, cereal cultivars had significant influence on fungal communities in their phyllospheres. On the other hand, disease resistances patterns at the cultivar level had no clear correlation with the fungal communities (Sapkota et al., 2015), which is inconsistent with our results. This difference could be attributed to the phyllosphere fungi samples from the previous studies containing endophytic and epiphytic fungi, which impacted community assembly (Yao et al., 2019). The combination of epiphytic and endophytic fungi probably prevented researchers from uncovering the true effect of disease resistance on the phyllosphere fungal community. Previous reports demonstrated that maize cultivar with different resistance to pathogenic fungi can structure the bacterial community on phyllosphere (Balint-Kurti et al., 2010). Our study found that both RDA and Linear Regression analysis showed that the disease index, which represents the resistance of maize to diseases, had the greatest impact on shaping the fungal communities. This suggests that the assembly pattern observed in the fungal community shaped by maize variety resembled that of the bacterial community. The resistance phenotype of a maize variety may directly affect the fungal community by suppressing or promoting some pathogenic maize fungi of maize to a certain extent. For example, *Exserohilum*, *Bipolaris*, *Cercospora* and *Curvularia* were only observed from the highly susceptible variety.

It is well known that plant genotypes determine leaf structure (such as cutin and cuticular wax properties) and leaf physiology (including leaf exudates and volatiles), which together significantly shape the microbial communities in a phyllosphere and microbe–microbe interactions (Bodenhausen et al., 2014; Horton et al., 2014; Farré-Armengol et al., 2016; Xin et al., 2016; Chen et al., 2020b). Principally, the leaf cell wall controls the quantity and quality of exudates and strongly influences on the microbe community of a phyllosphere. The absence of a cuticular membrane in single gene mutants of *Arabidopsis thaliana* dramatically affected the phyllosphere microbiota (Bodenhausen et al., 2014). The maize resistant gene *Htn1* against *E. turcicum* infection, encodes a cell wall-associated receptor-like kinase that regulates the cell wall structure (Kohorn and Kohorn, 2012; Hurni et al., 2015), and changes the chemical substances secreted by the leaves. Therefore, we measured the main chemical constituents in leaves, which were nitrogen, phosphorus and soluble sugar as well as the two secondary metabolites tannin and flavonoid. The HS and S varieties leaves had higher nitrogen content, which is related to the nitrogen-induced susceptibility to plant pathogen (Huang et al., 2017). RDA revealed that nitrogen was closely associated with the fungal communities of the S and HS varieties, also demonstrating that high nitrogen levels in S and HS varieties shape their fungal communities. This aligns well with the fact that phyllosphere bacteria are also influenced by nitrogen in susceptible varieties (Tian et al., 2020). Sugars determine the total microbial population in the phyllosphere (van der Wal and Leveau, 2011). We found that soluble sugars played minor roles in structuring the phyllosphere fungal communities of all four maize varieties, probably due to the absence of marked differences in soluble sugar contents in the leaves of the four maize varieties.

The HR variety had high phosphorus concentrations in the leaves when compared to the other varieties and this closely correlated with the fungal community in the HR leaf phyllosphere, suggesting that phosphorus affects the fungal communities. It is well known that phosphorus can promote plant tolerance to biotic and abiotic stresses (Pan, 2004), and our findings suggest that to some extent, high phosphorus levels are linked to high infection resistance. Phosphorus is also a key factor in shaping the microbes on a leaf surface (Yadav et al., 2005). An epiphytic microbe growth is limited by the availability of phosphorus (Schonherr and Baur, 1996), as it is difficult for ATP and polar P-containing compounds to penetrate the cuticles. Therefore, a high phosphorus concentration in the HR variety leaves provides a suitable environment for fungi and promote fungal growth.

Tannins and flavonoid contents also positively correlated with the fungal communities in the phyllosphere of HR and R varieties. Tannins are the most abundant secondary metabolites produced by plants and assist leaves in defending against insect herbivores by deterrence and/or toxicity (Barbehenn and Constabel, 2011). Flavonoids are also a vital secondary metabolite synthesized by plants and have important biological activity (Lewis and Ausubel, 2006; Song et al., 2021). Thus, the high content of tannins and flavonoids in the HR and R varieties also enhances their resistance to pathogens, and also regulates the fungal community in the phyllosphere. Two-factor correlation network analysis in this study revealed that flavonoids and tannins could alter the composition of the fungal community by specifically promoting or inhibiting certain types of fungi. As different plant varieties exhibit specific host attributes such as resistance, and higher nitrogen, potassium or phosphorus concentrations (Bodenhausen et al., 2014; Kembel and Mueller, 2014; Kembel et al., 2014), we speculated that the resistance of these maize varieties and their leaf chemical substances jointly shape the fungal community in the phyllosphere, which is in concordance with the view that the microbial community in a phyllosphere is controlled by multiple factors (Bokulich et al., 2014).

Microbial co-occurrence networks display the interaction between different species in a community (Deng et al., 2012). In this study, the composition of the fungal communities among the four maize varieties was similar, while the network structure was different, which indicated differences in the organization of the fungal community. Similar results were also found for microbes in the maize phyllosphere (Kong et al., 2020), which demonstrated that different maize varieties had different networks of microbial communities. We found that the complexity of the fungal community networks for the HR variety was relatively higher than the other three varieties, which indicated higher stability of the fungal community. This is supported by the notion that a resistant plant genotype frequently possessed a more complex microbial network in the rhizophsere and phyllosphere (Zhong et al., 2019; Xu et al., 2020). More positive associations in the HR fungal community included more cross feeding, co-aggregation, co-colonization and niche overlap in the community (Faust and Raes, 2012), suggesting a relatively healthy community. More negative relationships in the network of the S and HS fungal communities reflected amensalism, competition and antagonism in the community (Faust and Raes, 2012), which probably resulted from the colonization of fungal pathogen *Exserohilum* that disturbed the balance of the community.

## Data availability statement

The datasets presented in this study can be found in online repositories. The names of the repository/repositories and accession number(s) can be found below: https://www.ncbi.nlm.nih.gov/genbank/, PRJNA871799.

## Author contributions

KL provided study design, data analysis, manuscript writing, and supervision. GZ provided collection of phyllosphere fungi. MC provided data analysis. XT provided study design, supervision, and manuscript writing. All authors contributed to the article and approved the submitted version.

## Supplementary material

Supplementary Figure 1 Rarefaction curve and Venn diagram of fungi taxa among the four maize varieties. **(A)** Rarefaction curve at OTU level. **(B)** Venn diagram of fungi taxa at class level. **(C)** Venn diagram of fungi taxa at genus level. HR represents highly resistant variety. R represents resistant variety. S represents susceptible variety. HS represents highly susceptible variety.

Supplementary Figure 2 The relative abundance of *Exserohilum* in the fungal communities of four maize varieties. Different letters on the columns indicate the significant difference among the four maize varieties. HR represents highly resistant variety. R represents resistant variety. S represents susceptible variety. HS represents highly susceptible variety.

Supplementary Figure 3 The disease index and content of chemical substances in leaves of four maize varieties. **(A**) Disease index. **(B)** Nitrogen. **(C)** Phosphorus. **(D)** Tannins. **(E)** Flavonoids. **(F)** Soluble sugars. HR represents highly resistant variety. R represents resistant variety. S represents susceptible variety. HS represents highly susceptible variety. Different letters on the columns indicate the significant difference among the four maize varieties.

Supplementary Figure 4 Comparison between the main properties of co-occurrence networks among the fungal communities of four maize varieties. **(A)** Degree.**(B)** Clustering. **(C)** Closeness centrality. **(D)** Degree centrality. Different letters on the columns indicate the significant difference among the four maize varieties. HR represents highly resistant variety. R represents resistant variety. S represents susceptible variety. HS represents highly susceptible variety.
