# Supplementary figures and images for: Effects of maize resistance and leaf chemical substances on the structure of phyllosphere fungal communities
Source: Front Plant Sci. 2023 Aug 14;14:1241055. doi: 10.3389/fpls.2023.1241055 (PMC10461017; doi:10.3389/fpls.2023.1241055)

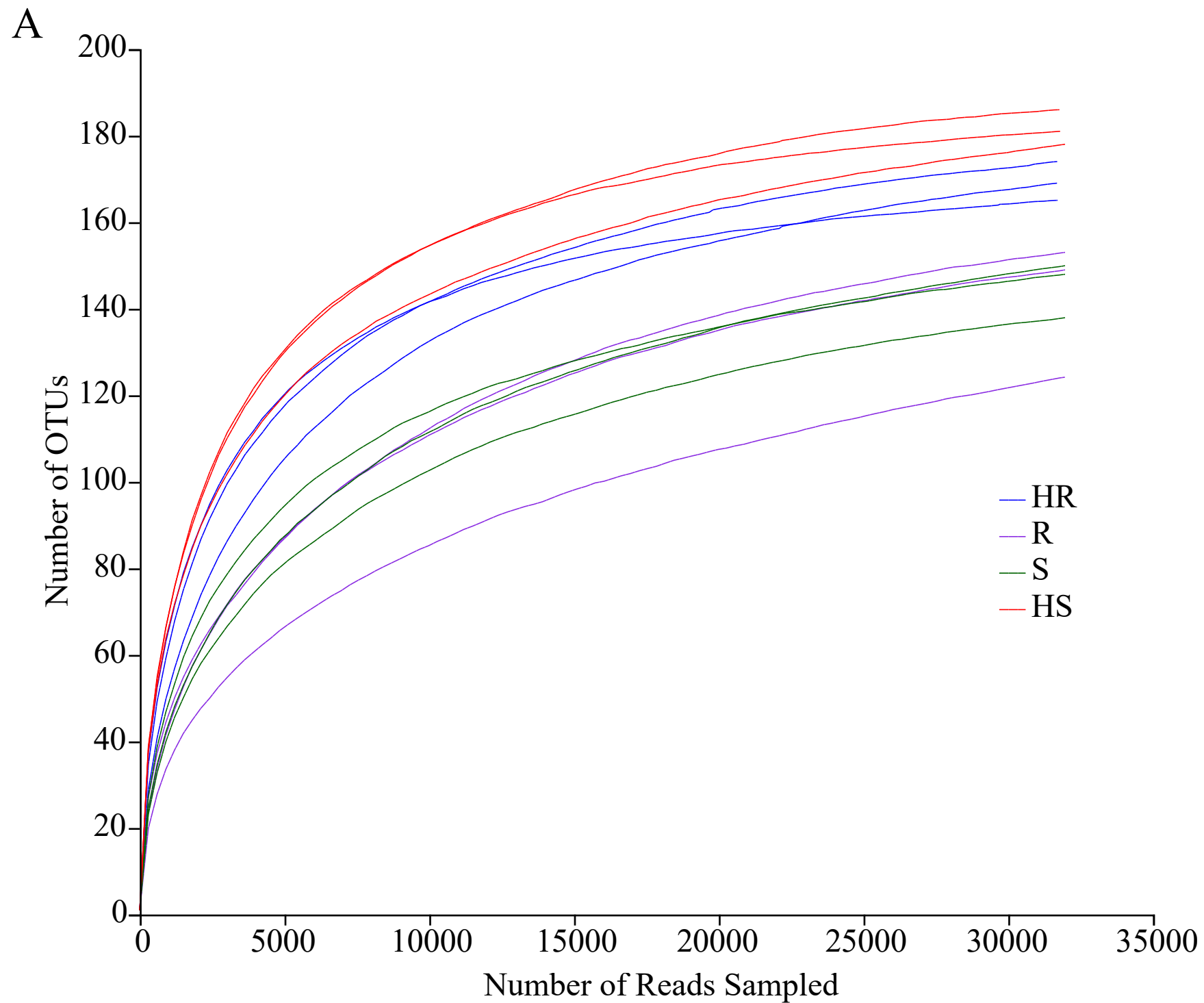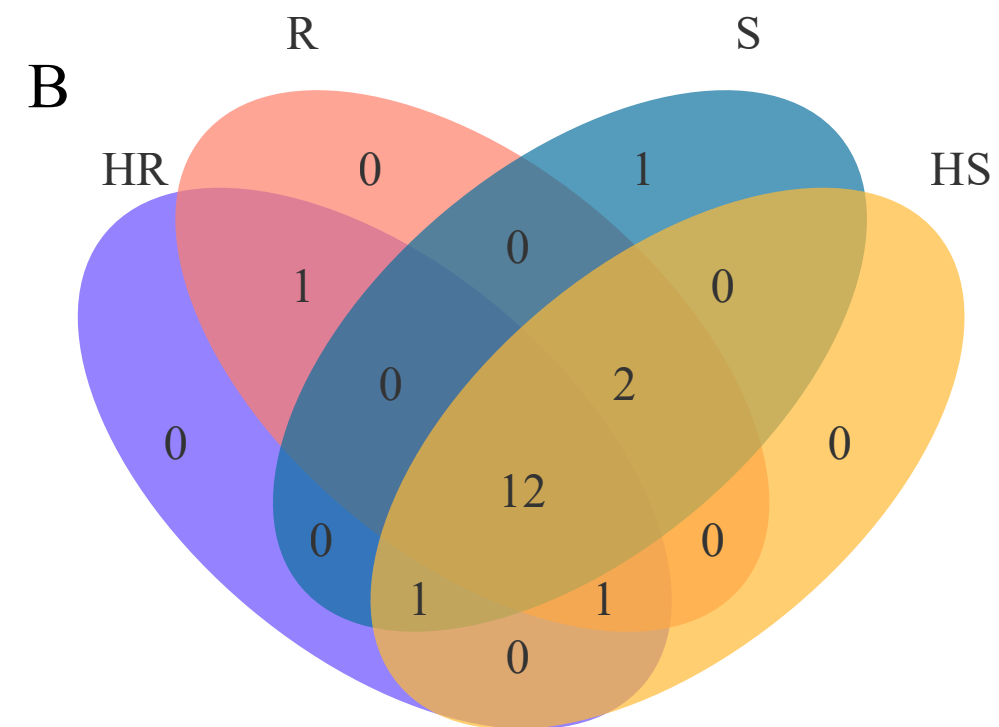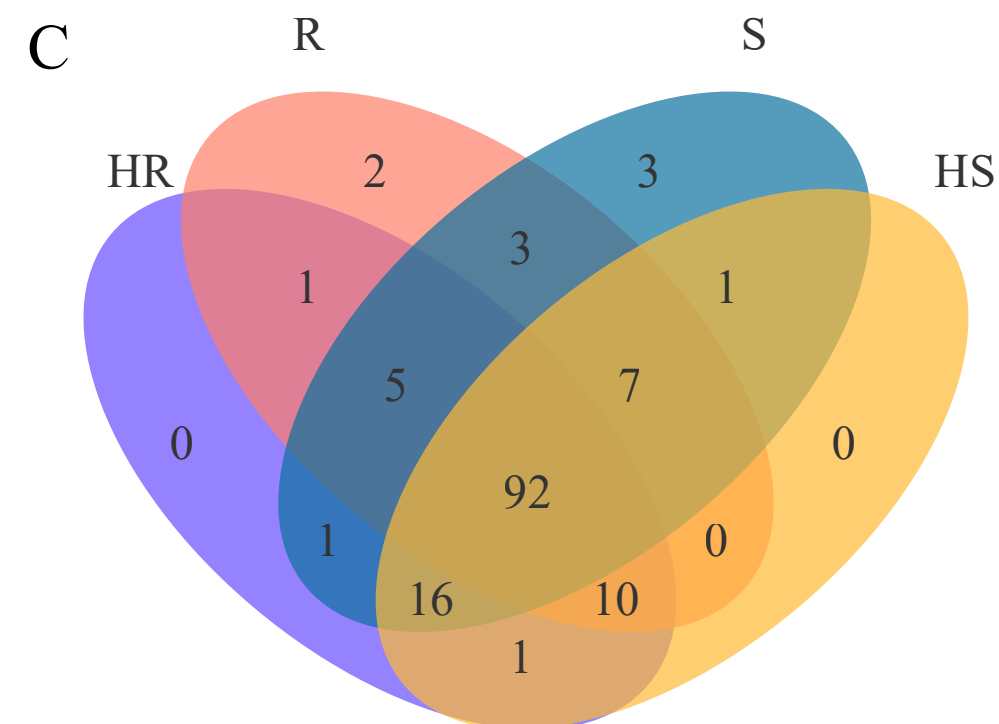

Supplement: Supplementary Figure 1 — Rarefaction curve and Venn diagram of fungi taxa among the four maize varieties. (A) Rarefaction curve at OTU level. (B) Venn diagram of fungi taxa at class level. (C) Venn diagram of fungi taxa at genus level. HR represents highly resistant variety. R represents resistant variety. S represents susceptible variety. HS represents highly susceptible variety. [file Image_1.pdf]

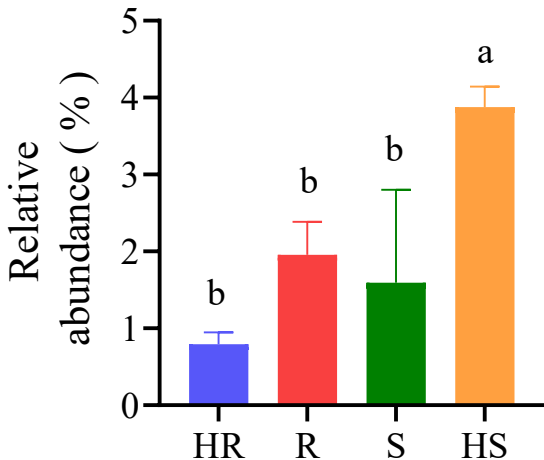

Supplement: Supplementary Figure 2 — The relative abundance of Exserohilum in the fungal communities of four maize varieties. Different letters on the columns indicate the significant difference among the four maize varieties. HR represents highly resistant variety. R represents resistant variety. S represents susceptible variety. HS represents highly susceptible variety. [file Image_2.pdf]

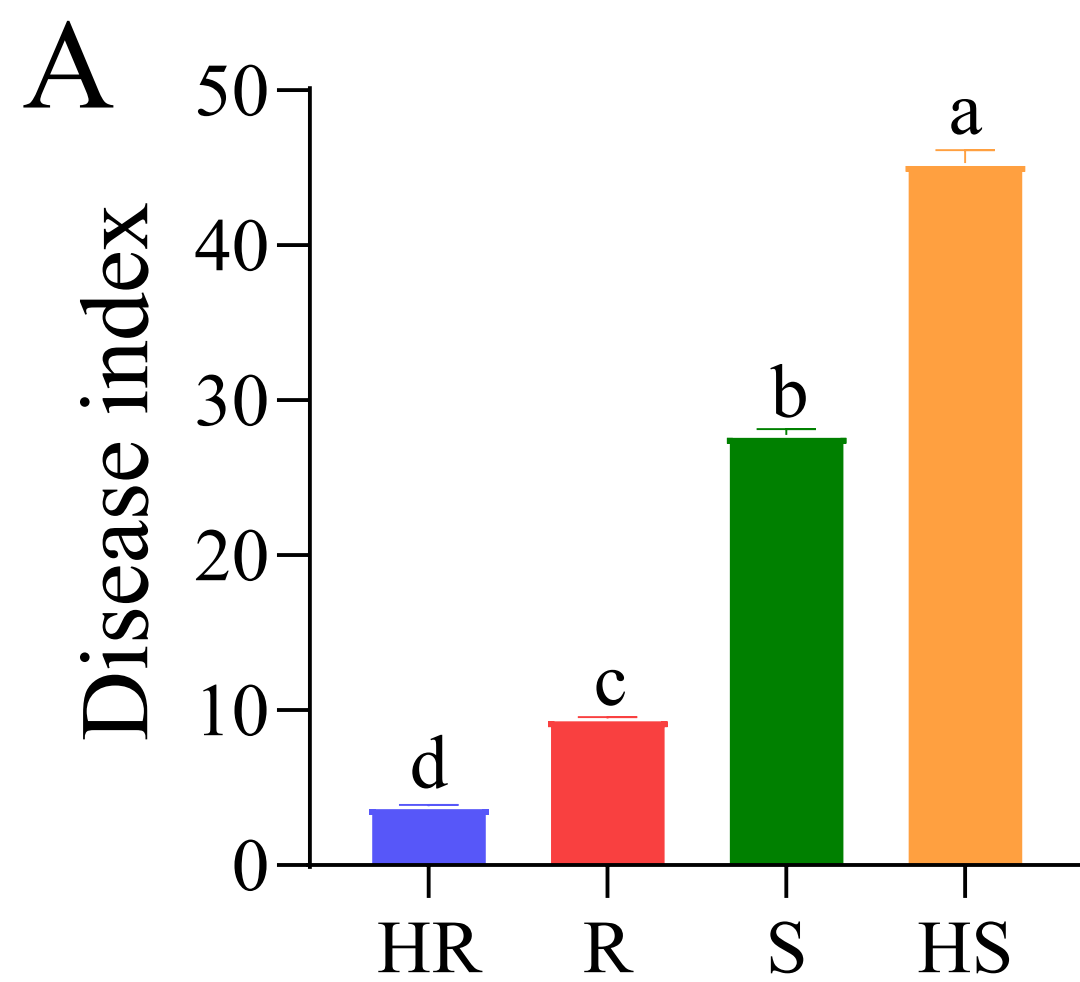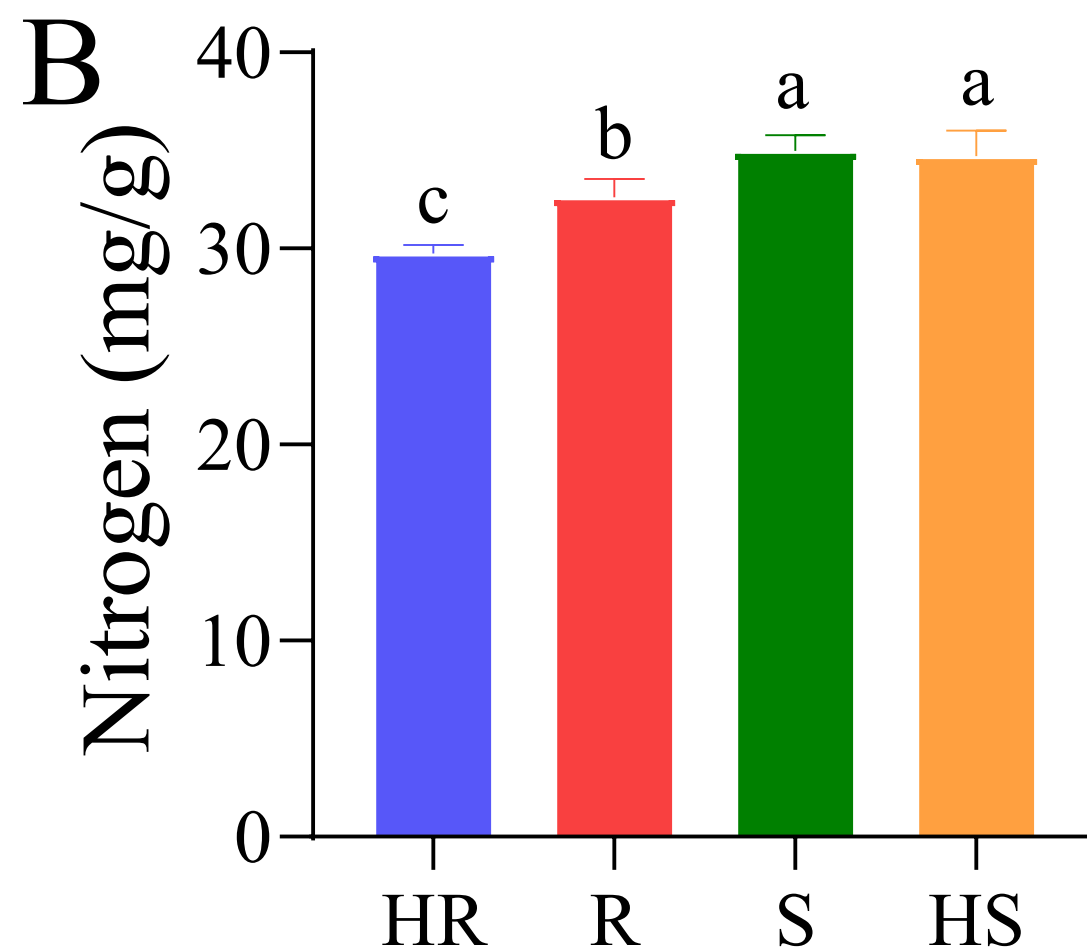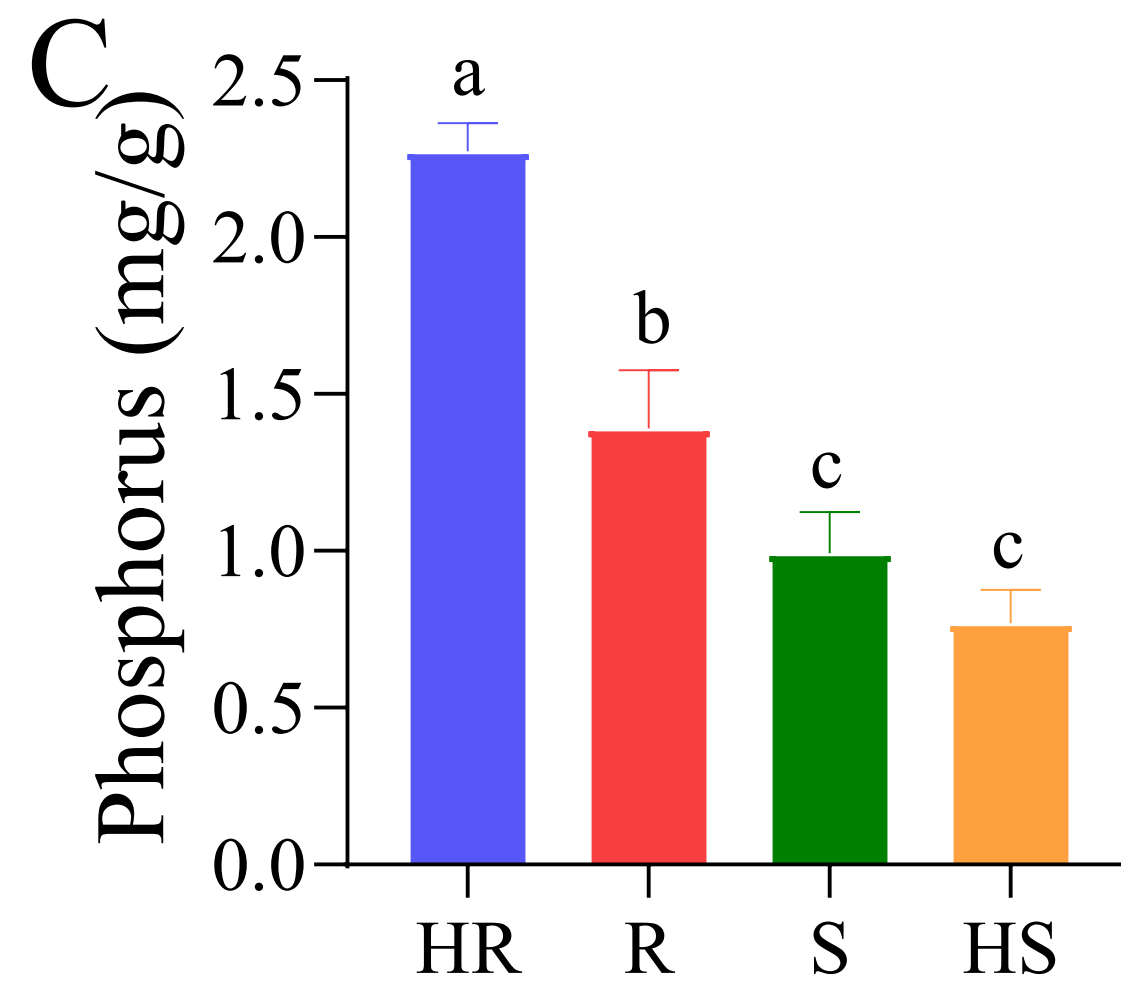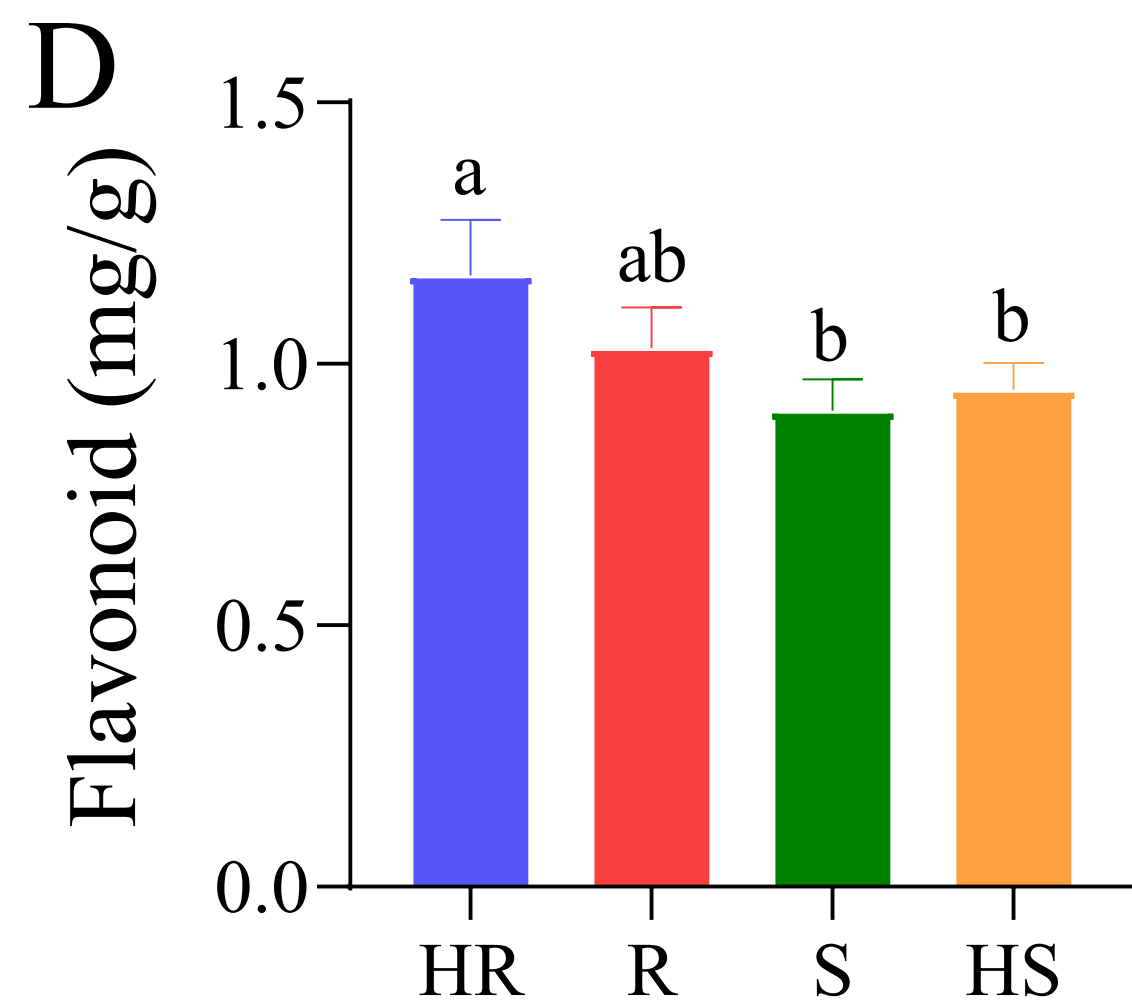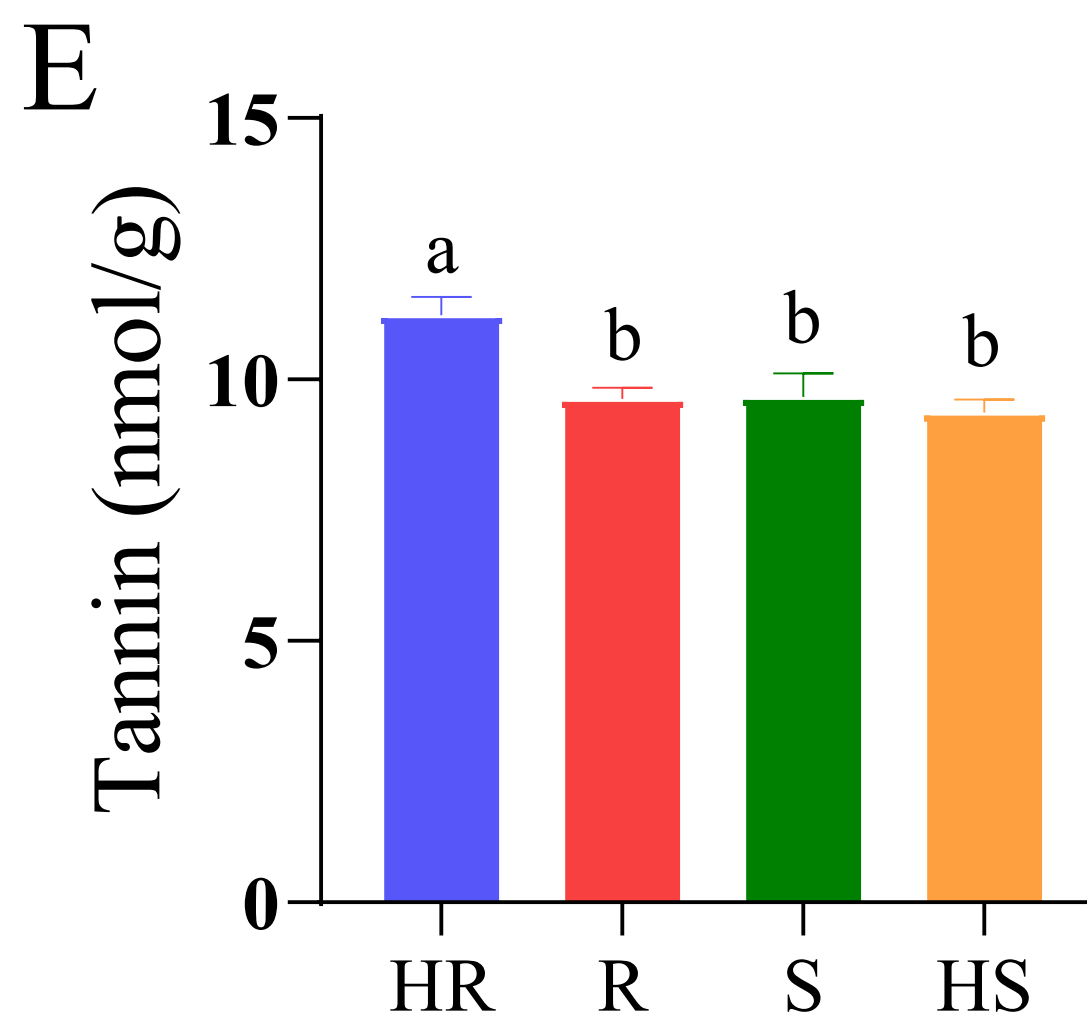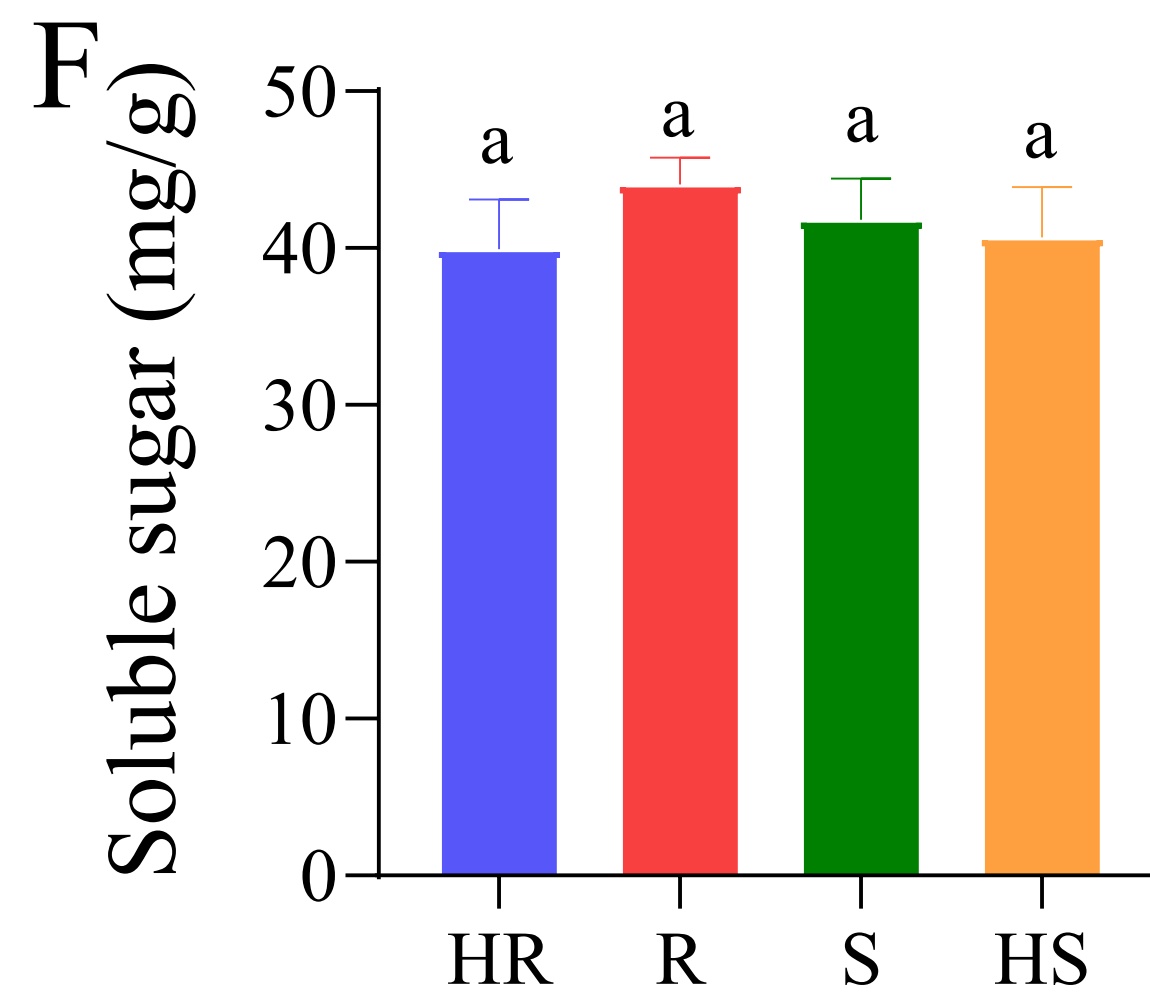

Supplement: Supplementary Figure 3 — The disease index and content of chemical substances in leaves of four maize varieties. (A) Disease index. (B) Nitrogen. (C) Phosphorus. (D) Tannins. (E) Flavonoids. (F) Soluble sugars. HR represents highly resistant variety. R represents resistant variety. S represents susceptible variety. HS represents highly susceptible variety. Different letters on the columns indicate the significant difference among the four maize varieties. [file Image_3.pdf]

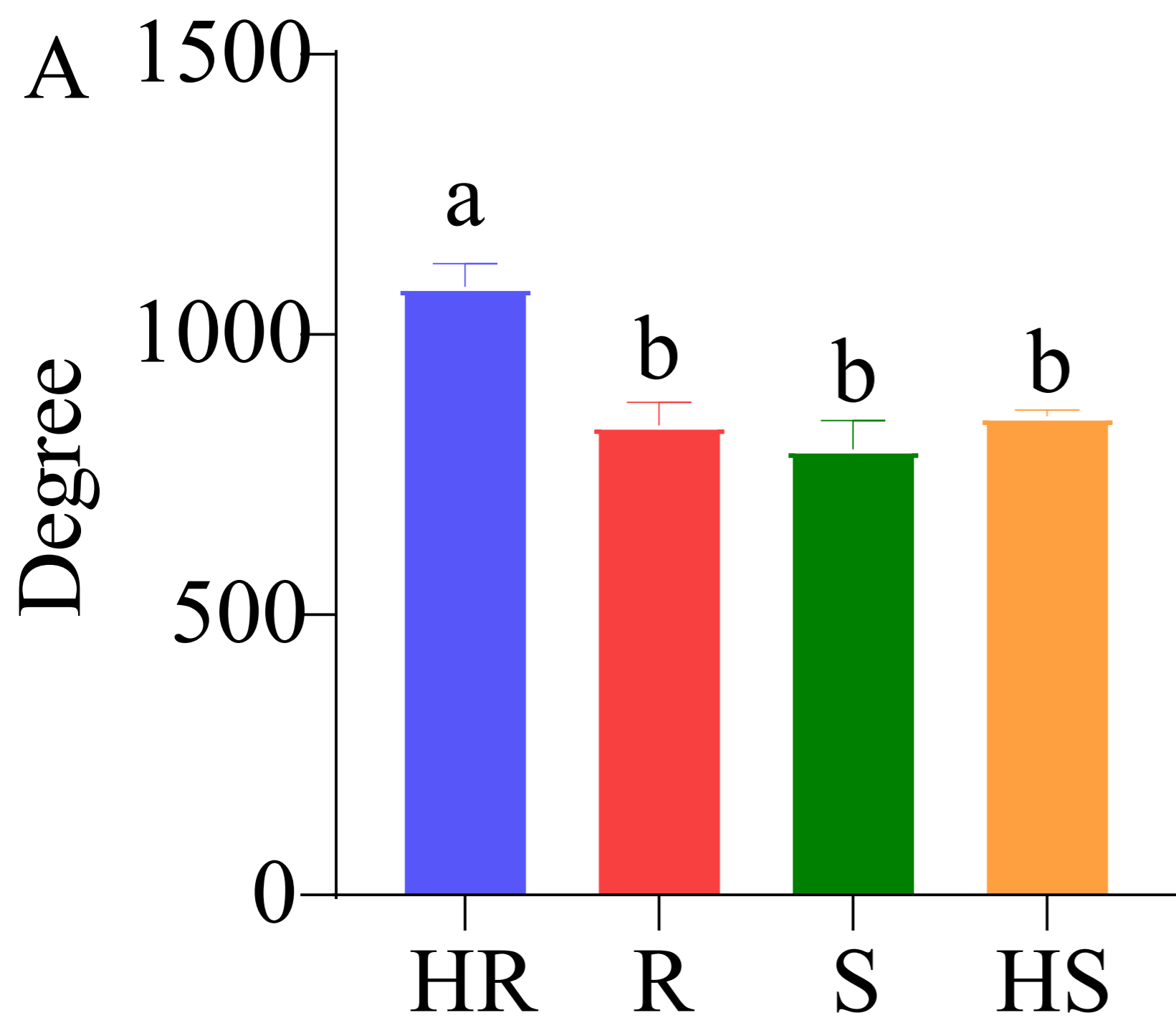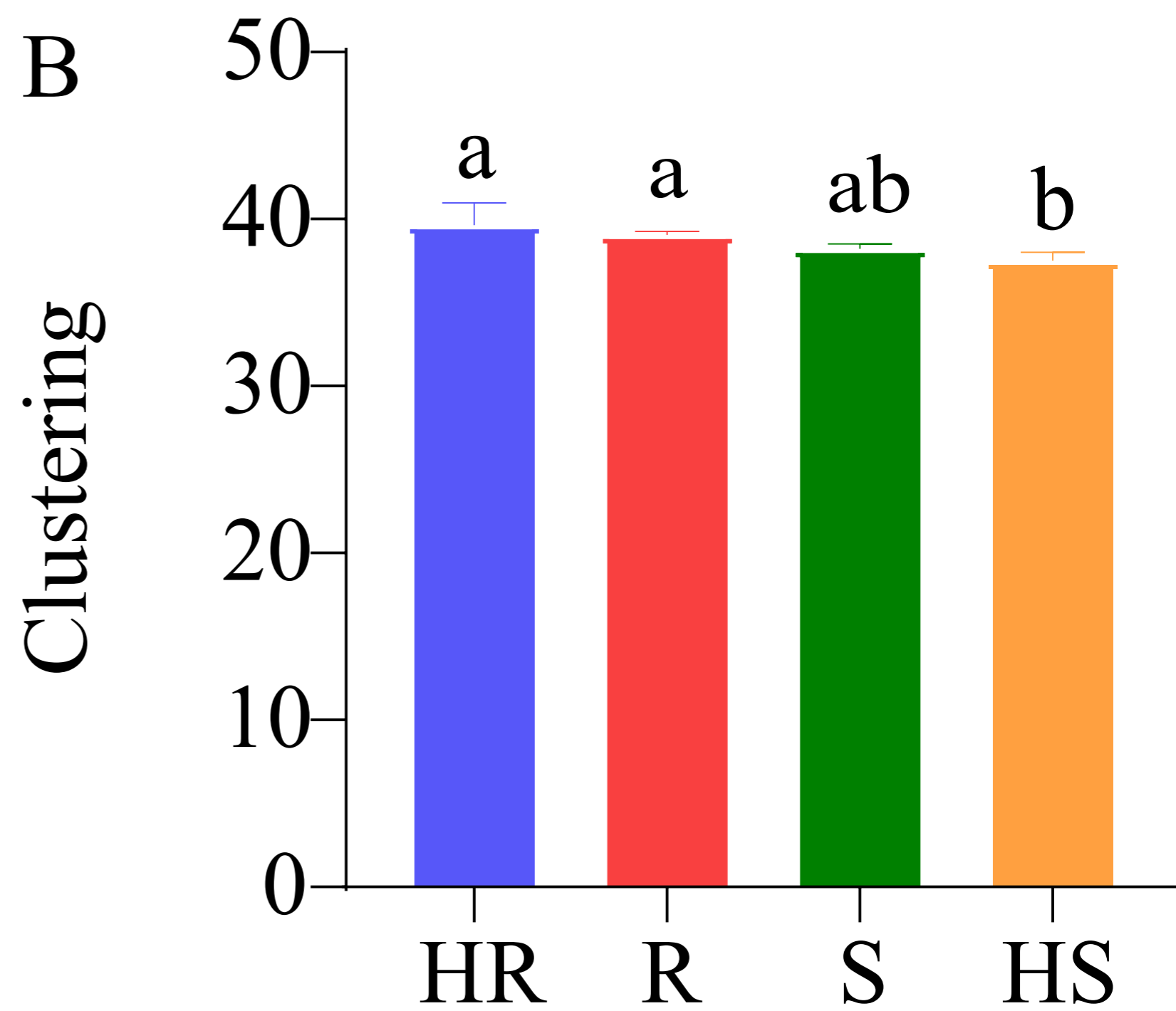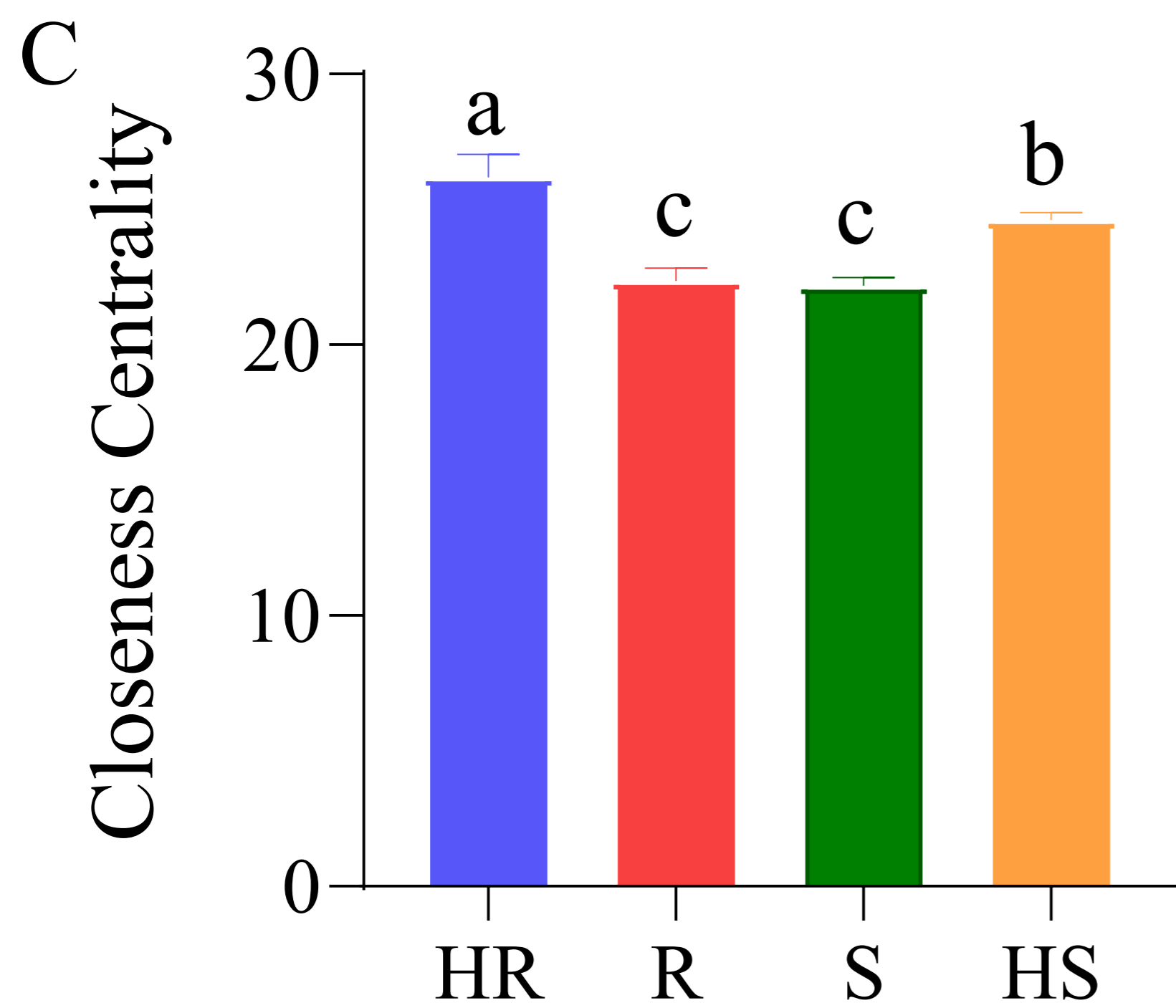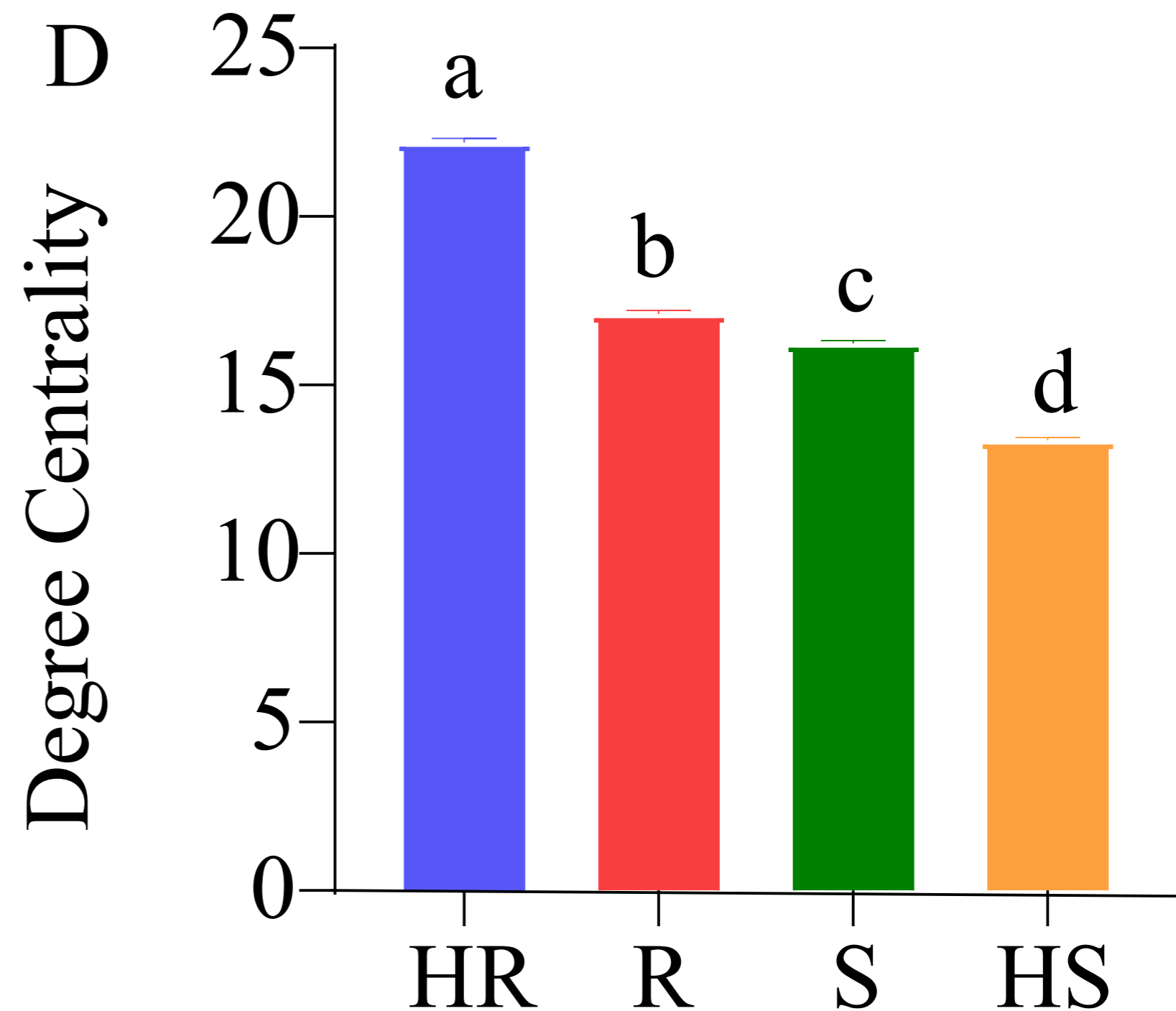

Supplement: Supplementary Figure 4 — Comparison between the main properties of co-occurrence networks among the fungal communities of four maize varieties. (A) Degree.(B) Clustering. (C) Closeness centrality. (D) Degree centrality. Different letters on the columns indicate the significant difference among the four maize varieties. HR represents highly resistant variety. R represents resistant variety. S represents susceptible variety. HS represents highly susceptible variety. [file Image_4.pdf]
