# Supplementary material for: Effects of maize resistance and leaf chemical substances on the structure of phyllosphere fungal communities
Source: Front Plant Sci. 2023 Aug 14;14:1241055. doi: 10.3389/fpls.2023.1241055 (PMC10461017; doi:10.3389/fpls.2023.1241055)
Supplement: Supplementary file 5 [file Table_1.docx]

**TABLE S1** Linear regression analyzed the relationship between disease index, leaf chemical constituents and the four fungal communities.

| leaf chemical constituents | R^2^ |
| --- | --- |
| Disease index | 0.927*** |
| Nitrogen | 0.690** |
| Phosphorus | 0.776*** |
| Soluble sugar | 0.055 |
| Flavonoid | 0.492* |
| Tannin | 0.620* |

R^2^ is the determination coefficient. Asterisks indicates significance (**P*<0.05; ***P*<0.01;****P*<0.001)
